# Therapy for advanced cholangiocarcinoma: Current knowledge and future potential

**DOI:** 10.1111/jcmm.16151

**Published:** 2020-12-04

**Authors:** Mingxun Wang, Ziyan Chen, Pengyi Guo, Yi Wang, Gang Chen

**Affiliations:** ^1^ Department of Hepatobiliary Surgery The First Affiliated Hospital, Wenzhou Medical University Wenzhou China; ^2^ Department of Cardiothoracic Surgery Ningbo Yinzhou NO.2 Hospital Ningbo China; ^3^ Department of Epidemiology and Biostatistics, Public Health and Management School Wenzhou Medical University Wenzhou China

**Keywords:** advanced tumour, cholangiocarcinoma, combination therapy, tumour therapy

## Abstract

Cholangiocarcinoma (CCA) is a biliary epithelial tumour that can emerge at any point in the biliary tree. It is commonly classified based on its anatomical site of development into intrahepatic cholangiocarcinoma (ICC), perihilar cholangiocarcinoma (PCC) and distal cholangiocarcinoma (DCC), each of which is associated with varying patient demographics, molecular characteristics and treatment options. CCA patients have poor overall prognoses and 5‐year survival rates. Additionally, CCA is often diagnosed at an advanced stage, with surgical treatment restricted to early‐stage disease. Owing to an increase in the incidence of ICC, that of CCA is also on the rise, with a corresponding increase in the associated mortality, particularly in South America and Asia. Therefore, the development of an effective treatment is crucial to improve the survival of CCA patients. We aimed to systematically review the current understanding of advanced CCA treatment and discuss potential effective strategies.

## 1 INTRODUCTION

**FIGURE 1:**
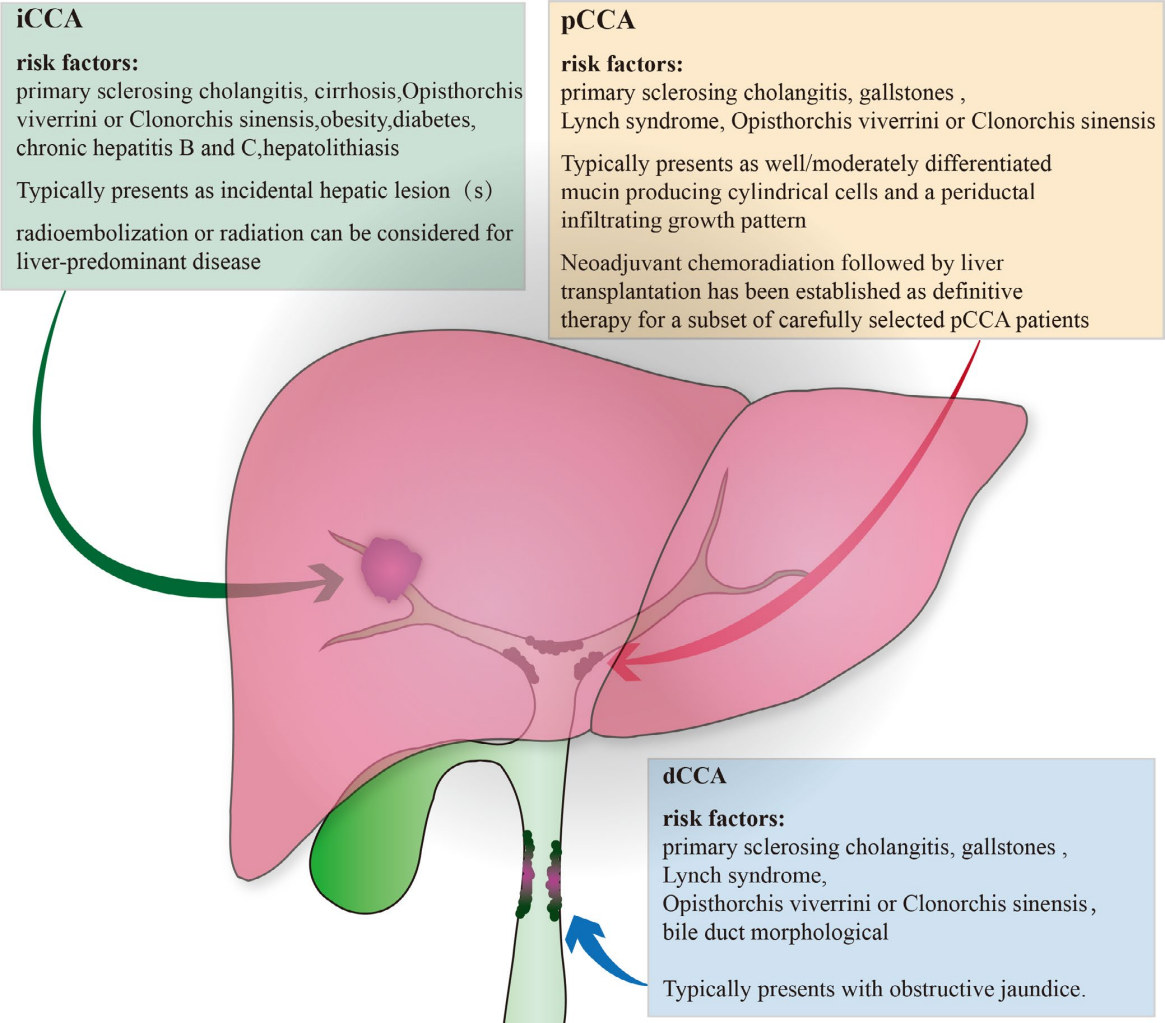
CCA is a heterogeneous disease that is classified into various subtypes including iCCA, pCCA and eCCA. The subtypes differ in many aspects, such as their anatomical location, risk factors, clinical presentations and treatment options

Cholangiocarcinoma (CCA) is the second most commonly occurring primary hepatobiliary cancer, accounting for 10%‐20% of all primary hepatic carcinomas.^1^, ^2^ The 5‐year relative survival rates range from 2% to 15% for intrahepatic cholangiocarcinoma (ICC) and 2%‐30% for extrahepatic cholangiocarcinoma (ECC).^3^ The majority of new CCA patients (60% ~ 70%) are diagnosed at a late stage and are treated with palliative therapy, particularly chemotherapy.^4^, ^5^ However, the prognoses remain unsatisfactory in such settings, despite the provision of first‐line chemotherapy. When a patient's condition deteriorates following first‐line chemotherapy, the recommended approach is supportive care.^6^ CCA has low sensitivity to chemotherapy, and only a few effective anticancer drugs are available for its treatment. Traditionally, the occurrence of CCA is considered rare; however, the global incidence rate of the disease, particularly ICC, has steadily increased over the past 15 years.^1^, ^7^ Cholangiocarcinoma is commonly classified based on its anatomical site of presentation into the ICC and ECC subtypes. ECC is further divided into perihilar cholangiocarcinoma (PCC) and distal cholangiocarcinoma (DCC), with each subtype showing different epidemiological, molecular and therapeutic characteristics (Figure 1). Although the aetiology of CCA has not been determined, several risk factors have been identified. For example, some risk factors for ICC include primary sclerosing cholangitis (PSC), liver cirrhosis, *Opisthorchis viverrine* infection and *Clonorchis sinensis* infection, whereas PSC, gallstones and Lynch syndrome are risk factors for ECC.^8^, ^9^, ^10^ Surgery is currently the most effective and preferred treatment option for CCA. However, surgical resection can be performed in only approximately 35% of patients with early‐stage disease. Besides, the rate of post‐operative recurrence is high even in patients with surgical resection.^11^, ^12^ For patients with advanced or unresectable CCA, the available systemic therapies have limited effectiveness, with gemcitabine and platinum demonstrating a median overall survival <1 year.^13^ Proliferative heterogeneity, genetic heterogeneity, and tumour microenvironment changes promote tumour progression and potentially hinder the effectiveness of chemotherapy. Therefore, in this review, we aimed to investigate the current developments and emerging concepts pertaining to the aforementioned therapies in CCA settings.

## 2 SYSTEMIC THERAPY

First‐line chemotherapy: gemcitabine and platinum are the most commonly used first‐line agents in CCA treatment.^13^, ^14^ The use of chemotherapy as a palliative treatment to improve patients' quality of life was first reported in a study in 1996, and gemcitabine was established as a possible treatment option for those with advanced disease.^15^ The findings of that study increased the level of research interest in the use of chemotherapy in CCA treatment. A phase III clinical trial (ABC‐02 trial) demonstrated that the efficacy of gemcitabine plus cisplatin (GC) was superior to that of gemcitabine alone.^13^ A randomized phase II trial focusing on the use of gemcitabine and cisplatin in Japan (BT22 trial) showed that the 1‐year survival rate was higher in the combination treatment group than in the gemcitabine group (31.0% in the gemcitabine group and 39.0% in the GC group).^16^ In another phase 2 clinical trial, gemcitabine plus nab‐paclitaxel was used as the first‐line treatment for advanced or metastatic cholangiocarcinoma. However, the trial did not meet its primary efficacy end‐point.^17^ Based on the aforementioned results, gemcitabine and platinum are still recognized as first‐line treatment options for patients with advanced or metastatic CCA.

Second‐line chemotherapy: currently, there is a lack of strong evidence supporting the use of second‐line chemotherapy in CCA.^18^ However, a few prospective and retrospective studies have shown that second‐line chemotherapy is effective in select CCA patients.^19^, ^20^, ^21^, ^22^, ^23^ First‐line treatment agents such as gemcitabine and cisplatin are associated with several side effects such as nausea, vomiting and anorexia, leading to hydration drug requirement and treatment resistance development. This illustrates the need for different and alternative treatment mechanisms. Fluoropyrimidines such as 5‐fluorouracil, capecitabine and S‐1 are commonly used in clinical practice. Second‐line chemotherapy, including the combination of mitomycin C and capecitabine vs. capecitabine alone, yielded a disappointing outcome, with the addition of mitomycin C failing to show improved results.^24^ A randomized phase II study focusing on the efficacy of the second‐line oxaliplatin plus irinotecan (XELIRI) regimen versus that of irinotecan monotherapy demonstrated a clear progression‐free survival (PFS) benefit.^25^ In addition, the sequence GEMOX‐based followed by FOLFIRI‐based chemotherapy resulted in the achievement of an overall survival (OS) duration of 21.9 months in 52 patients.^26^

## 3 RADIOTHERAPY

The role of radiotherapy in the treatment of primary liver cancer was previously limited. However, recent developments in the treatment design have allowed for the consideration of radiotherapy as a potential treatment modality. Traditionally, the most effective treatment for CCA, when feasible, is surgical resection; the use of liver transplantation remains controversial.^27^ However, in some patients with ICC or ECC, the disease is locally advanced or unresectable, predominantly owing to local vascular invasion or inadequate baseline hepatobiliary function. Therefore, non‐surgical treatments have elicited widespread interest, with several such treatments having emerged, including novel systemic therapy, ethanol ablation, transarterial chemoembolization, radiofrequency ablation and stereotactic body radiation therapy (SBRT).^28^, ^29^ High‐resolution, multiphase spiral computed tomography (CT) and multi‐parameter magnetic resonance imaging can accurately determine the cancer location and radiotherapy target range. Furthermore, CT‐based treatment planning and dose calculation can aid in the accurate estimation of the radiation dose delivered to the tumour and non‐malignant tissues.^30^, ^31^ SBRT delivers high doses of radiation with an elevated degree of precision, thereby minimizing the radiation therapy dose to the adjacent normal tissues.^32^, ^33^ In particular, SBRT has attracted interest as a feasible and non‐invasive treatment method. This treatment can be used for advanced or unresectable primary hepatobiliary cancer.^34^, ^35^ In addition, selective internal radiotherapy (SIRT) plus chemotherapy or hepatic arterial infusion plus systemic chemotherapy both had encouraging efficacy and are promising for the treatment of advanced cholangiocarcinoma.^36^, ^37^ These advances may enhance the efficacy of radiation therapy for CCA as well as improve the level of protection of non‐malignant tissues, thereby enhancing the efficacy of radiation treatment in CCA patients.

## 4 TARGETING THE MOLECULAR BIOLOGY OF CCA

### 4.1 Development of molecular‐targeted therapy

There is currently a lack of an effective targeted therapy for CCA, owing to the presence of significant inter‐tumour and intra‐tumour heterogeneity. Moreover, in a majority of clinical trials, researchers classify patients with different subtypes of CCA based on a broad definition of the disease rather than gene specific carcinogenic drivers. Today, through molecular profiling studies, it is possible to understand, in detail, the genomic and transcriptomic phenotypes of each CCA subtype. There are significant differences in the prevalence of oncogenic mutations between ICC and ECC, suggesting that the oncogenic processes of these tumour subtypes are different (Table 1). Besides, the prevalence of mutations is highly variable across different studies, potentially in association with regional differences, small sample sizes, or variations in the pathologic classification of ICC and ECC before sequencing. In a study by Nakamura et al, comprehensive exome and transcriptome sequencing were performed in 260 patients (145 patients with ICC, 86 with ECC and 29 with gallbladder carcinoma). Approximately 40% of the patients showed genetic changes that could be targeted,^38^ and all the components associated with the genetic change varied across the different CCA subtypes. For instance, recurrent mutations in isocitrate dehydrogenase (IDH)1/2, fibroblast growth factor receptor (FGFR)1‐3 and BAP1 were primarily present in ICC, whereas mutations in ARID1B, ELF3 and PRKACB occurred predominantly in ECC; the characteristics associated with the different genetic aberrations in each disease subtype contributed to its unique biological behaviour. The fusion of FGFR2 resulted in the ligand‐independent activation of receptor tyrosine kinase, which occurs only in patients with ICC,^38^ consistent with previous findings.^39^, ^40^, ^41^, ^42^ The new gene fusion involved PRKACA or PRKACB, which encodes a catalytic subunit, and a protein kinase A, which has only been detected in ECC. The identification of these aberrations is important, as gene fusions are recognized as important driver genes. ELF3 is another driver gene that is found primarily in ECC.^38^ Therefore, ETS‐related transcription factor—ELF3—may act as an inhibitor of CCA,^43^ consistent with the findings of Nakamura et al In previous studies, targeted sequencing was performed on select cancer‐related genes including IDH1/2, FGFR2 and CDKN2A; the most significant changes were reported in ARID1A, IDH1/2 and TP53 (each of which was found in 36% of the tumours) and MCL1 (amplified in 21% of the tumours).^38^, ^40^

**Table 1:** Molecular aberrations in CCA

	ECC	ICC	Reference
TP53 mutation	40%	2.5%‐44.4%	56, 105, 106
ERBB2 amplification	11%‐17%	3%	38, 105
HER2 overexpression	5%‐20%	0%‐2%	40, 74, 76
CDKN2A/B loss	17%	5.6%‐25.9%	40, 105
ARID1A mutation	12%	6.9%‐36%	40, 57, 67, 105, 107
KRAS mutation	40%‐47%	8.6%‐24.2%	56, 105, 108
PIK3CA mutation	9%	4%‐6%	40, 109
BRAF mutation	6%	4%‐22%	42, 56
VEGF overexpression	59%	54%	40, 74, 76
FBXW7 mutation	15%	6%	40
NF1 mutation	NR	4%	40
BRCA1/2 mutation	NR	4%	40
TSC1 deletion	NR	4%	40
SMAD 4 mutation	11%‐25%	1%‐4%	105, 106, 107
EGFR overexpression	5%‐19%	11%‐27%	40, 74, 76
IDH1/2 mutation	0%‐7.4%	4.9%‐36%	56, 63, 107
MET overexpression	NR	7%‐21%	40, 74

Abbreviation: NR, not reported.

In patients with CCA, indirect carcinogenic exposure may cause significant somatic changes. The total exome sequencing of 108 liver fluke‐associated tumours and 101 non‐liver fluke‐associated tumours revealed^44^ that the incidence of IDH1 or IDH2 mutations was higher in cases of ICC unrelated to hepatic fluke, which also led to the deletion of the tumour suppressor gene BAP1. However, mutations in the tumour suppressor gene TP53 were associated with a higher incidence of hepatic trematode CCA.^44^ These findings suggest that different aetiologies determine the phenotype of mutant CCA. The Cancer Genome Atlas performed a comprehensive genomic analysis of liver fluke‐negative and hepatitis‐negative ICC, and reported the presence of inactivating mutations in tumour suppressor genes ARID1A, ARID1B, TP53 and PTEN, and functional mutations in oncogenes IDH1/2, BRAF and KRAS.^45^ Consistent with previous studies, IDH1 or IDH2 mutations were detected and expressed only in the ICC subtypes.^38^, ^40^

### 4.2 Emerging molecular targeting therapy

**FIGURE 2:**
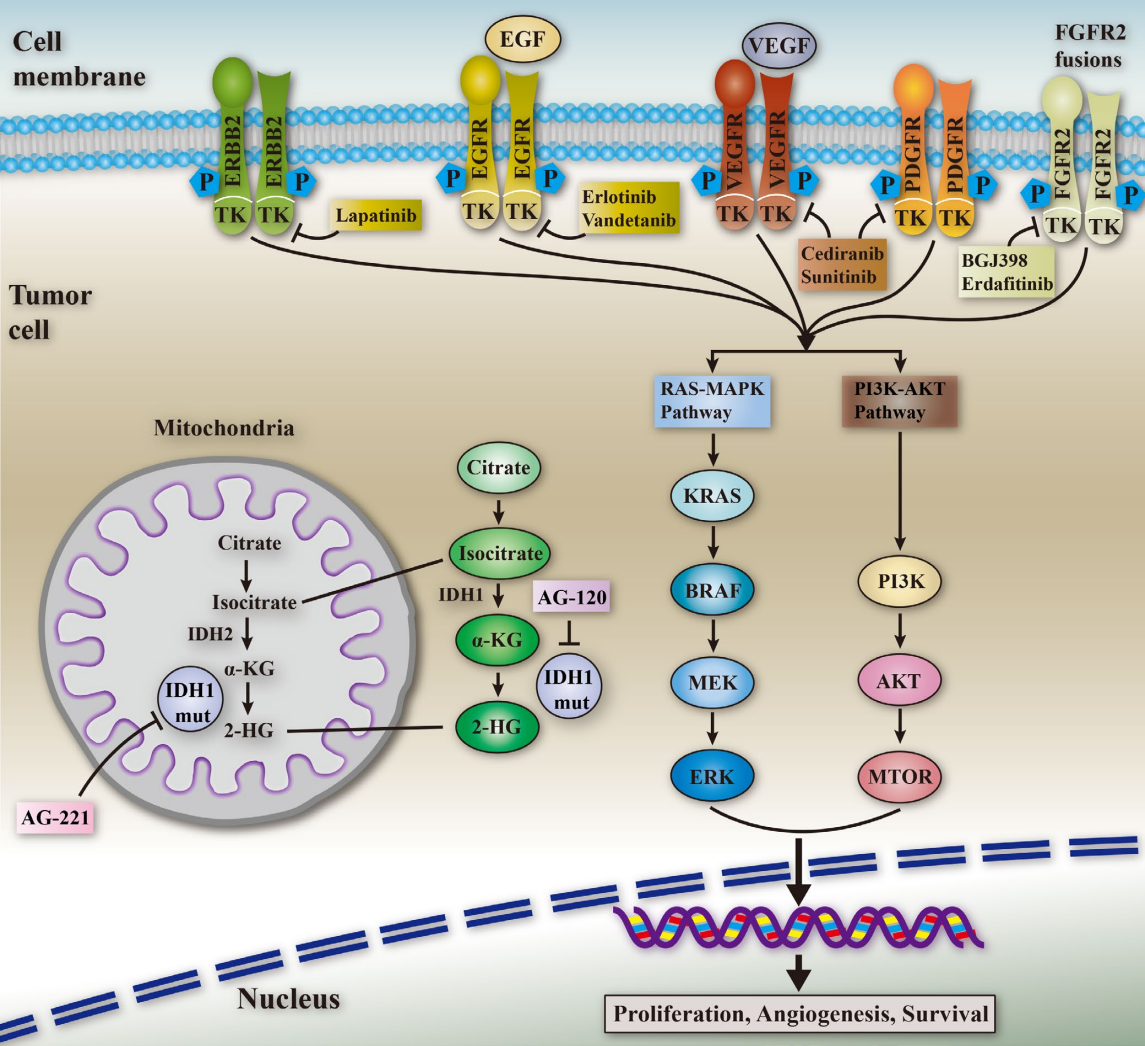
Key signalling pathways in the pathogenesis of cholangiocarcinoma and relative targeted agents. Genetic alterations in tyrosine kinase receptors and downstream RAS/MAPK, PI3K/AKT/mTOR effector cascades result in aberrant modulation of proliferation, apoptosis and cell cycle. Mutated IDH1/IDH2 result in the accumulation of oncogenic metabolite 2HG. Potential targeted agents against the relative alterations are also shown. CCA, cholangiocarcinoma; ICC, intrahepatic cholangiocarcinoma; ECC, extrahepatic cholangiocarcinoma; pCCA, perihilar cholangiocarcinoma; dCCA, distal cholangiocarcinoma; PSC, primary sclerosing cholangitis; GC, gemcitabine plus cisplatin; GS, gemcitabine plus S‐1; SBRT, stereotactic systemic circulation therapy; CT, computerized tomography; MRI, magnetic resonance imaging; FGFR, fibroblast growth factor receptor; IDH, isocitrate dehydrogenase; TCGA, The Cancer Genome Atlas; 2‐HG, 2‐hydroxyglutaric acid; TK, tyrosine kinase; EGFR, epidermal growth factor receptor; OS, overall survival; PFS, progression‐free survival; PADGF, platelet activity‐derived growth factor; WT‐1, Wilms tumour‐1; Muc‐1, mucin protein 1; TAF, tumour‐associated fibroblasts; TAM, tumour‐associated macrophages; CTLA‐4, cytotoxic T lymphocyte‐associated protein 4; PD‐1, programmed death 1; PD‐l1, programmed death‐ligand 1; MSI, microsatellite instability; MMR, mismatch repair; SIRT, selective internal radiotherapy; ORR, overall response rate

Some recent studies identified key carcinogenic drivers as promising targets and, accordingly, tailored compounds to those targets (Figure 2). The efficacy of erlotinib,^46^ cetuximab,^47^ panzumab,^48^ sorafenib,^49^ silenib^50^ and van der thanil^51^ was evaluated in some randomized trials of molecularly targeted drugs. A multi‐centre, open‐label, randomized phase Ⅲ study reported that the addition of erlotinib to chemotherapy extended the median PFA in patients with CCA (median 5.9 months [95% confidence interval [CI] = 4.7‐7.1] vs. 3.0 months [1.1‐4.9]; hazard ratio = 0.73, 95% CI = 0.53‐1.00; *P* = .049).^46^ They were all designed to evaluate the effectiveness of cytotoxic drugs such as GEMOX, 5‐fluorouracil, GC and gemcitabine. The erlotinib evaluation study was a phase III trial. Currently, no molecular‐targeted drugs have been proven as being effective against advanced CCA.

#### 4.2.1 Isocitrate dehydrogenase 1 and 2 mutation

Isocitrate dehydrogenase promotes the conversion of isocitrate to α‐ketoglutarate and participates in the citric acid cycle and other metabolic processes.^52^, ^53^, ^54^ When IDH undergoes mutation, the rate of metabolites that produce 2‐hydroxyglutaric acid (2‐HG) increases, causing extensive epigenetic changes that affect the rates of cell differentiation, growth and hypoxia signalling.^55^ IDH1 mutations have been observed in 7%‐36% of ICC cases.^56^, ^57^, ^58^, ^59^ In a study that focused on the use of AG‐120 (ivosidenib) in 73 patients with IDH1‐mutated ICC, 5% of the patients showed partial response to the diagnosis and 56% experienced stable disease, and the 6‐month PFS rate was 40%.^60^ Subsequently, a phase III, placebo‐controlled trial was conducted in patients with previously treated IDH1‐mutant CCA. This study demonstrated that patients had an increased PFS in the experiment group, which was 2.7 months compared to 1.4 months in the control group.^61^

Although IDH1/2 mutations are relatively commonly reported in gliomas, they were previously thought to have a low incidence in other malignancies.^62^ However, several studies have shown that IDH mutations occur relatively frequently in CCA.^56^, ^60^ Furthermore, IDH mutations are more commonly reported in ICC than in PCC and DCC (23%‐28% vs 0%‐7%).^58^, ^62^ In a histopathological analysis of surgically resected primary CCA tissue, IDH1/2 mutations were shown to be associated with significant cellular changes and poor tissue differentiation.^62^ Additionally, patients with IDH1/2 mutations who underwent surgery also showed better 1‐year overall survival rates than patients without IDH mutations.^62^ The presence of a positive correlation between IDH mutations and prognoses was also observed in a study of 326 ICC cases. In that study, IDH mutations were shown to profoundly affect the ICC patients' OS and tumour recurrence rates after surgical resection.^63^ Other studies have concluded that the association between IDH mutations and prognoses requires further investigation. Jiao et al evaluated 34 ICC registration groups and showed that the 3‐year survival rate in patients with IDH gene mutations was 33%, whereas the corresponding value in those with wild‐type IDH gene mutations was 81%. However, patients with IDH gene mutations are characterized by older age and a higher tumour grade.^57^ An analysis of somatic mutations in 200 resected ICC tissues found that IDH1 mutations were the most commonly occurring type. However, IDH1 mutational status does not seem to affect patients' long‐term prognoses.^56^ Although the aforementioned studies showed heterogeneous results, they predominantly focused on early or resectable ICC, while targeted therapy with IDH inhibitors was considered for unresectable or advanced ICC.^64^ A study that evaluated the correlation between IDH mutations and prognoses in 104 patients with unresectable or advanced ICC found that IDH mutations did not significantly affect the median OS.^64^

#### 4.2.2 FGFR2 fusion

FGFR2, a cell surface receptor for fibroblast growth factors, is located on chromosome 10q26.^65^ It plays a key role in the regulation of cell proliferation, differentiation, survival, wound repair, angiogenesis and migration.^66^ Recently, with the use of whole‐exome sequencing and fluorescence in situ hybridization, multiple FGFR2 chromosome fusions with genomic partners in some cancers, including ICC, have been identified.^67^ The tumorigenic activity of FGFR2 fusion proteins is dependent on activation, and the rearranged tyrosine kinases influence dimerization, subsequent tyrosine autophosphorylation and increased downstream signalling.^42^ The carcinogenic potential of trans‐FGFR2 fusions both in vitro and in vivo, such as FGFR2‐AHCYL1,^68^ FGFR2‐TACC3,^40^ FGFR2‐MGEA5^67^ and FGFR2‐BICC1^41^ has been previously reported on. Furthermore, the presence of FGFR2 fusion seems to indicate a more sensitive and selective target for FGFR2 inhibition.^69^ However, both the relative tumorigenic potential of different FGFR2 fusion proteins and their sensitivity to specific FGFR2 inhibitors require more in‐depth research. Screening for FGFR2 fusion proteins previously demonstrated significant differences in the incidence of FGFR2 fusion proteins, ranging from 3% to 50% in ICC patients. The incidence of FGFR2 fusion is low in mixed hepatocellular carcinoma (HCC)‐ICC and is largely absent in HCC and ECC.^41^, ^42^ The recent results of the multi‐centre, open‐label, phase II FIGHT‐202 trial showed that the overall response rate (ORR) was 35.5% with a median of 9.1 months of the duration of response.^70^ On the basis of positive results, the US FDA has approved the anti‐FGFR2 antibody pemigatinib in 10‐16% of patients with cholangiocarcinoma harbouring FGFR2 gene fusions. Therefore, FGFR2 fusion can be considered a promising marker for ICC.

The presence of FGFR2 fusion with KRAS mutation and signalling pathway activation suggests its possible synergistic role in the pathogenesis of ICC. In a Japanese study, a remarkable link with viral hepatitis was found, whereas in a study conducted in North America, a significant association was found among women.^42^ However, further studies are needed to confirm these findings. In addition to FGFR2 fusion, 8.7% of CCA tumour tissues have the ROS1 kinase fusion protein. The expression of fused‐in‐glioblastoma‐c‐ros‐oncogene (FIG–ROS)1 can be transformed both in vitro and in vivo, and can be specifically inhibited.^71^ The carcinogenicity of FIG‐ROS has recently been demonstrated in KRAS and TP53 mutations in orthotopic allograft mouse models of ICC.^72^ An ongoing phase II trial of infigratinib—an oral FGFR inhibitor for advanced CCA with FGFR abnormalities (gene fusion, translocation or other genetic alterations)—showed interesting results, with an overall response rate of 14.8% (FGFR2 fusion present only at a response rate of 18.8%).^73^ Preliminary data support the effectiveness of ROS1 kinase in the targeting of small ATP competitive inhibitors both in vitro and in vivo. Further investigations are needed to determine the frequency of ROS fusion in different patient populations with ICC and assess the potential benefits of this therapy for translocated alleles.

#### 4.2.3 Epidermal growth factor receptor/human epidermal growth factor receptor 2

Human epidermal growth factor receptor 2 (HER2) is a member of the epidermal growth factor receptor (EGFR) family and is associated with the multi‐step carcinogenesis of CCA.^74^, ^75^ Dimerization of the receptor results in the autophosphorylation of tyrosine residues and initiates the downstream signalling pathways that regulate cell proliferation and tumorigenesis. Several studies that focused on the mutation profiling of CCA specimens identified EGFR overexpression in 11%‐27% of ICCs and 5%‐19% of PCCs/DCCs.^74^, ^76^ EGFR overexpression in CCA is predominantly characterized (77%‐79% of cases) by an increased copy number, and the activation of EGFR mutations is extremely rare.^74^ Although no mutation was observed in HER2, its overexpression was found in 0%‐2% of ICC patients and 5%‐20% of PCC/DCC patients. Several phase II clinical trials have combined cetuximab with other chemotherapy agents, particularly gemcitabine and oxaliplatin, in the treatment of CCA.^77^, ^78^, ^79^ In a previous phase II study, the addition of cetuximab, a chimeric anti‐EGFR monoclonal antibody, to gemcitabine‐oxaliplatin did not yield a survival benefit in patients with advanced CCA. The median PFS associated with gemcitabine‐oxaliplatin plus cetuximab was 6.1 months, whereas the median OS was 11 months; the corresponding values for gemcitabine plus oxaliplatin alone were 5.5 months and 12.4 months, respectively.^80^ In a study in which patients were stratified according to their KRAS status and received GEMOX with or without cetuximab,^47^ the combination regimen of cetuximab, gemcitabine and oxaliplatin yielded improved median PFS values (6.7 months vs. 4.1 months; *P* = .05); the effect did not extend to the median OS value (10.6 months vs. 9.8 months; *P* = .91). Moreover, KRAS mutations do not predict survival. Therefore, additional biomarker‐driven trials must be performed to provide further insights, as KRAS mutation has not been suggested as a predictive biomarker of the EGFR response to treatment.

#### 4.2.4 Vascular endothelial growth factor

The most relevant pro‐angiogenic protein that is associated with tumour growth and metastasis is vascular endothelial growth factor (VEGF).^76^, ^81^ VEGF expression is associated with several poor prognostic characteristics, distant ICC metastasis and increased microvascular density in CCA. Microvascular density is an independent prognostic factor for disease‐free survival after ECC resection and an independent prognostic factor for OS in node‐negative ICC. It is also an independent negative predictor of OS in ECC. Several clinical trials have evaluated the effects of VEGF inhibitors. In a phase II trial focusing on bevacizumab in combination with gemcitabine and oxaliplatin, fluorodeoxyglucose‐positron emission tomography demonstrated a significant reduction in the standard intake after two treatment cycles, especially in patients with partial remission or disease stabilization.^82^ However, the 6‐month PFS (63%) was below the target rate of 70%. The combination of bevacizumab and erlotinib (an anti‐EGFR tyrosine kinase inhibitor) yielded partial response in 12% of the patients and 51% of the patients showed stable disease, with a median OS of 9.9 months, which was notable due to the lack of use of concurrent chemotherapy.^83^

## 5 IMMUNOTHERAPY

The association between chronic inflammation and CCA development has allowed for the control of the level of immune response through immunization, acquired immunotherapy and checkpoint suppression. Limited results have been observed in vaccination studies with single‐drug therapy, with the most commonly observed targets being WT‐1 and muc‐1. Wt‐1 is a transcription factor that acts as a tumour suppressor through interactions with platelet‐derived growth factor receptor, EGFR, c‐myc and bcl‐2. Phase I studies focusing on the WT‐1 vaccine in combination with gemcitabine showed that patients with T cell response to the WT‐1 vaccine had longer OS durations than those treated with gemcitabine alone.^84^ Muc‐1 is a glycoprotein that forms a hydrophilic barrier, which inhibits the uptake of hydrophobic cytotoxic substances and immune surveillance. It is highly overexpressed in gallbladder cancer (90%), with lower expression levels in CCA (77%), and is associated with advanced cancer survival. An earlier study showed that despite the presence of immunoglobulin G response, the mut‐1 vaccine did not yield clinical benefits.^85^ Therefore, it may be meaningful to expand the vaccine to two,^86^ three^87^ or four^88^ peptides, or even develop a personalized peptide vaccine^89^; studies focusing on the same are in the early stages. Shimizu et al vaccinated patients in whom CCA removal was achieved using autologous tumour lysate‐pulsed dendritic cells plus ex vivo activated T cell transfer.^90^ The OS of these patients (31.9 vs. 17.4 months, *P* = .022) was almost twice as high as that among those who underwent surgery alone; the effect was particularly significant in patients with skin reactions.^91^

As new evidence shows, the use of immune checkpoint inhibitors is among the most promising approaches for improving the efficiency of cancer immunotherapy.^92^, ^93^, ^94^ T cells predominantly assume the function of regulation in the immune system. CCA has a rich tumour microenvironment including cancer‐associated fibroblasts, tumour‐associated macrophages and lymphocytes.^95^, ^96^, ^97^ In the tumour microenvironment, T cell response can be fine‐tuned by immune checkpoints and regulated through the signals sent by T cell receptors. They can be excitatory or inhibitory and participate in the different T cell response stages.^98^, ^99^ Many cancers evade the immune system primarily by the overexpression of the inhibitory ligands that inhibit T cell attack. Cytotoxic T lymphocyte‐associated protein 4 (CLAT‐4) predominantly regulates T cell tolerance and has become a major focus in immunotherapy. Anti‐CLAT‐4 monoclonal antibodies have also been considered in clinical cancer therapy and are highly effective in multiple cancers. Additionally, programmed death protein 1 (PD‐l) and PD‐1 ligand (PD‐L1) inhibitors are being used in clinical cancer immunotherapy. The United States Food and Drug Administration has approved the use of anti‐PD‐1 antibodies (pembrolizumab) for previously treated patients with DNA mismatch repair (MMR) defects and/or microsatellite instability. Notably, 5 to 10% of CCA patients are reported to have MMR defects. Pembrolizumab is a highly selective, humanized PD‐1 monoclonal antibody that is designed to block the interaction between PD‐1 and its ligands—PD‐L1, and PD‐L2. In a study of 260 patients with CCA, 45% of those who underwent molecular identification showed immune checkpoint molecule up‐regulation.^38^ The expression of PD‐1/PD‐L1 was shown to be up‐regulated in a group of different ICCs.^100^ Although the dysregulation of immune checkpoint molecules is associated with a lower degree of histologic differentiation, a more advanced tumour stage and poor prognoses, human leucocyte antigen 1 overexpression seems to have an inverse effect.^101^

## 6 CONCLUSION

Despite its low incidence, CCA has attracted increased attention due to its lethality.^102^ Currently, the standard first‐line chemotherapeutic regimen for advanced CCA is GC therapy; however, a standard regimen in the second‐line setting has not yet been established. Although the efficacy of molecular targeting agents has been satisfactory so far, attractive genetic mutations have also been reported. Future developments in genomic screening methods using high‐throughput sequencing are expected. A recent retrospective study showed that the blocking of HER‐2/neu is a promising treatment approach in patients with CCA.^103^ Immunotherapy has previously yielded promising results in patients with recurrent, refractory and heavily pre‐treated CCA. New therapeutic methods are increasingly being used in the treatment of advanced and extended tumours, such as photodynamic therapy (PDT). Recent studies have found that the OS duration associated with PDT combined with chemotherapy is significantly longer than that related to chemotherapy alone (*P* = .022).^104^ Therefore, as a larger body of data on these combinations become available, the survival and treatment outcomes of CCA patients are expected to improve further. Future studies should continue to pay close attention to therapies that target specific genetic aberrations. A comprehensive genome map could also help in the identification of treatment options and provision of personalized treatment for patients with advanced CCA. Through the optimization of the personalized treatment provided to CCA patients, the currently bleak outcomes are expected to significantly improve.

## CONFLICT OF INTEREST

The authors confirm that there are no conflicts of interest.

## AUTHOR CONTRIBUTIONS

**Mingxun Wang:** Investigation (equal); Visualization (equal); Writing‐original draft (equal); Writing‐review & editing (equal). **Ziyan Chen:**; Investigation (equal); Visualization (equal); Writing‐original draft (equal); Writing‐review & editing (lead). **pengyi guo:** Investigation (equal); Validation (equal); Visualization (supporting); Writing‐review & editing (supporting). **Yi Wang:** Conceptualization (lead); Funding acquisition (equal); Investigation (equal); Project administration (lead); Visualization (lead); Writing‐original draft (lead); Writing‐review & editing (lead). **Gang Chen:** Conceptualization (equal); Funding acquisition (equal); Project administration (equal); Writing‐review & editing (equal).
